# High flow nasal therapy in perioperative medicine: from operating room to general ward

**DOI:** 10.1186/s12871-018-0623-4

**Published:** 2018-11-10

**Authors:** Andrea Cortegiani, Giuseppe Accurso, Sebastiano Mercadante, Antonino Giarratano, Cesare Gregoretti

**Affiliations:** 1 Department of Surgical, Oncological and Oral Science (Di.Chir.On.S.). Section of Anesthesia, Analgesia, Intensive Care and Emergency. Policlinico Paolo Giaccone, University of Palermo, Via del vespro 129, 90127 Palermo, Italy; 2 Anesthesia and Intensive Care and Pain Relief and Palliative Care Unit, La Maddalena Cancer Center, Palermo, Italy

**Keywords:** High flow nasal therapy, Noninvasive ventilation, Acute respiratory failure, Perioperative medicine

## Abstract

**Background:**

High flow nasal therapy (HFNT) is a technique in which humidified and heated gas is delivered to the airways through the nose via small nasal prongs at flows that are higher than the rates generally applied during conventional oxygen therapy. The delivered high flow rates combine mixtures of air and oxygen and enable different inspired oxygen fractions ranging from 0.21 to 1. HFNT is increasingly used in critically ill adult patients, especially hypoxemic patients in different clinical settings.

**Main body:**

Noninvasive ventilation delivers positive pressure (end-expiratory and inspiratory pressures or continuous positive airway pressure) via different external interfaces. In contrast, HFNT produces different physiological effects that are only partially linked to the generation of expiratory positive airway pressure. HFNT and noninvasive ventilation (NIV) are interesting non-invasive supports in perioperative medicine. HFNT exhibits some advantages compared to NIV because HFNT is easier to apply and requires a lower nursing workload. Tolerance of HFNT remains a matter of intense debate, and it may be related to selected parameters. Patients receiving HFNT and their respiratory patterns should be closely monitored to avoid delays in intubation despite correct oxygenation parameters.

**Conclusion:**

HFNT seems to be an interesting noninvasive support in perioperative medicine. The present review provides anesthesiologists with an overview of current evidence and practical advice on the application of HFNT in perioperative medicine in adult patients.

## Background

High flow nasal therapy (HFNT) is a technique in which humidified and heated gas is delivered to the airways through the nose via nasal prongs, which are larger than conventional nasal prongs, at higher flow rates than are generally applied during conventional oxygen therapy. Inspiratory high flow of humidified air may be used alone or in combination with oxygen to generate different inspiratory oxygen fractions (FiO_2_) ranging from 0.21 to 1. Noninvasive ventilation (NIV) delivers noninvasive continuous positive airway pressure (CPAP) or noninvasive positive end expiratory pressure (PEEP) plus an inspiratory pressure to the patient’s airway via different external interfaces [1]. HFNT produces different physiological effects that are only partially linked to the generation of an end expiratory airway pressure [2].

HFNT is receiving growing attention as an alternative respiratory support in critical care settings, and it is used in patients with different underlying diseases. [2–8]. Several studies suggest that HFNT decreases a patient’s effort and respiratory rate and eventually reduces the need for more invasive respiratory support in patients with different lung diseases [2–7]. It has been also demonstrated that HFNT may be a potential alternative to NIV, especially in patients with acute hypoxemic respiratory failure who exhibit a ratio of arterial blood and delivered oxygen (PaO_2_/FiO_2_) < 200 [9, 10]. Currently, the evidence on the use of NIV in perioperative medicine is growing [6, 11–13]. There are several reasons why HFNT may be an alternative or complementary to NIV in perioperative medicine 1) NIV applies high continuous or intermittent positive pressure [14], and it remains the best noninvasive tool to increase functional residual capacity (FRC) [1, 15]. However, HFNT exhibits some unique physiological mechanisms that are discussed in this review. 2) NIV is not always applicable because of technical aspects, such as the patient’s poor tolerance to the interface during NIV and problems fitting the mask to the patient [16, 17]. 3) Effective application of NIV presupposes specific knowledge and training. However, inadequate use of HFNT may delay intubation, which was observed during the early years of NIV [18, 19].

This review aims to provide anesthesiologists with an overview of current evidence and practical advice on the application of HFNT in perioperative medicine in adult patients.

## Main text

### What is high flow nasal therapy?

Devices that offer high flows of totally humidified gases (37 °C, 100% relative humidity (RH), 44 mg H_2_O/L) from 20 to 60 L/min through specific nasal cannulae are commonly called HFNT systems. A heated humidifier connected to a heated insulated single-limb circuit provides active humidification. High flow may be generated in different ways:

1. Using a Venturi system driven by high oxygen pressure. The predetermined FiO_2_ is obtained from a blender. The lowest applicable FiO_2_ is 0.3 because the system is driven only by oxygen;
2. Using two high pressure sources, namely, high pressure air and oxygen. The predetermined FiO_2_ is obtained according to the total amount of oxygen divided by the total delivered flow (i.e., HFNT 60 L/min: 50 L/min of air and 10 L/min of oxygen; 50 L/min of air contains 21% of oxygen equal to approximately 10 L/min; if this 10 L/min extrapolated from the 50 L/min of air are added to 10 L/min of oxygen, the total amount of oxygen will be approximately 20 L/min; if the overall flow is 60 L/min (10 plus 50 L/min) the FiO_2_ will be 20 × 100/60, equal to a FiO_2_ close to 0.33);
3. Using a turbine system. A turbine allows the delivery of heated and humidified gases up to 60 L/min. FiO_2_ is predetermined only if the turbine has an internal blender. Alternatively, a low-pressure oxygen inlet allows the addition of oxygen up to 60 L/min with a dedicated flow meter. An oxygen cell determines the FiO_2_;
4. Using a conventional compressed air or turbine-driven mechanical ventilator with a dedicated HFNT system. FiO_2_ is predetermined by the ventilator blender [20].

Several mechanisms explain how HFNT reduces dyspnea and improves arterial blood gases and patient comfort [2, 21]:

A) *Improvement in the oxygen pharyngeal concentration in patients undergoing treatment with oxygen.*

Oxygen administration is the first-line supportive treatment in patients with acute hypoxemic respiratory failure. The maintenance of adequate oxygenation depends on the management of FiO_2_. Oxygen is generally administered via facial masks or nasal prongs at a flow that generally does not exceed 15 L/min. FiO_2_ pharyngeal values are unstable using conventional oxygen therapy in the presence of patients with high inspiratory flows and respiratory rates [14]. By limiting air entrainment, HFNT might maintain high FiO_2_ levels via the application of high flow rates above a patient’s inspiratory requirements [2, 22, 23]. Chanques et al. did not find significant differences in FiO_2_ between mouth closed and open when HFNT was applied at 45 L/min at FiO_2_ 1 [14]. In a bench study, administration of oxygen through an HFNT system showed no lack of performance at different inspiratory tidal volume and respiratory rates [23].

B) *Less metabolic cost for gas conditioning.*

Inspired air is heated to 37 °C and humidified to 100% RH during normal breathing [24]. This process involves an energy cost [25] that can be reduced by the use of HFNT or an optimal humidification system during NIV [25]. Theoretically, each liter of inspired air requires 107.5 joules (26 cal) to increase the temperature and humidity of room air, based on ambient air at room temperature (21 °C) and 50% humidity [25]. A normal adult breathing a 500-ml tidal volume for each breath at a respiratory frequency of 12 breaths/min requires approximately 156 cal/min for the heating and humidifying of inspired gas [25]. The metabolic cost of heating inspiratory gases increases in patients who generate higher minute ventilation due to acute respiratory failure (ARF). HFNT may also affect carbon dioxide (CO_2_) production by decreasing the energy expenditure for conditioning gas [25].

C) *Reduction of inspiratory resistances.*

During inspiration, the negative pressure generated by the respiratory muscles may cause a retraction of the nasopharynx [26] that may increase inspiratory resistance of the upper airway. HFNT can reduce respiratory resistance by 1) meeting or exceeding the patient’s peak inspiratory flow by supplying gas at a high flow; 2) “splinting” the nostrils by activating the nasal muscles [27, 28]; and 3) reducing bronchoconstriction from the nasal inhalation of cold air [28, 29].

D) *Improvement in mucociliary clearance.*

Practice guidelines recommend conditioning oxygen when its delivery exceeds 4 L/min, but patients undergoing oxygen therapy usually receive very low conditioned gas because optimal humidification cannot be reached by the nasal mucosa [30]. Critically ill patients may benefit from HFNT because it improves the humidification of inspired gases to decrease the symptoms of dryness and eventually improve comfort [31]. Breathing cold and low conditioned gas may also modify the mucociliary transport system [32]. HFNT can also improves airway humidification and possibly improves mucus clearance retention [33].

E) *Increased expiratory resistance.*

During physiological inspiration the pressure inside the nostrils becomes negative, but the pressure at the beginning and during most of inspiration remains above atmospheric pressure using HFNT because of an increase in expiratory resistances. Two major factors may play a role in increasing expiratory resistance and pressure, primarily a given EPAP level [34]:

1) An increase in expiratory resistance per se. Expiratory resistance is generated by the in-going jet flow against exhaled expiratory air and the size of the cannula [34]. Nasal cannula size plays an important role in controlling EPAP levels during HFNT because EPAP level depends on the number of leaks around the small prongs. The size of the nasal cannula is appropriate when it occupies approximately 2/3 of the surface of the nostrils [35];

2) Mouth closed or mouth open. A significant difference in generated EPAPs between mouth open and mouth closed was demonstrated [14]. Opening the mouth, independently from the inspiratory flow decreased mean airway pressure from 2 to 0.6 cmH_2_O [14]. This decrease is a crucial point because most patients with acute respiratory failure breathe with their mouth open. Furthermore, an increase in expiratory resistance may decrease respiratory rate via an increase in expiratory time (inspiratory time remains unchanged), which is similar to the respiratory model of the pursued lips breathing (PLB) that is often adopted by patients with chronic obstructive pulmonary disease (COPD) [36, 37].

It is important to stress that HFNT does not provide CPAP [38]. Nevertheless, although the pressure inside the pharynx may be lower than during CPAP because the interface used during HFNT is not tightly fitted to the nostrils, [14], this pressure may be sufficient to increase the end-expiratory lung volume (EELV) [39].

F) *Nasopharyngeal dead space washout.*

At the beginning of inspiration, nasopharyngeal dead space encloses the end-expiratory gases. The dead space contributes to the heating and optimal humidification of the inspired air and decreases the efficiency of gas exchange. By reducing the dead space inside the nasopharynx via the insufflation of fresh gas, HFNT decreased the overall dead space and improves alveolar ventilation [37, 40]. In a bench study, HFNT, delivered at 30 L/min, eliminated CO_2_ contained in the dead space of the nose model during the very beginning of expiration and swapped it with fresh air [40]. Therefore, patients with COPD or lung fibrosis may require a lower increase in alveolar ventilation to reduce CO_2_ [41–43].

Interestingly, although HFNT is often termed high flow oxygen therapy, most of its beneficial effects can be achieved at FiO_2_ 0.21, as observed during CPAP.

Flow and FIO_2_ should be titrated separately during HFNT application. FiO_2_ titration is important in patients with COPD because over-oxygenation may cause hypercapnia. Flow should be set as high as possible, up to 60 L/min, to match the patient’s inspiratory flow [44]. Temperature is also a very important issue. HFNT temperature seems to significantly impact the comfort of patients with hypoxemic ARF. Mauri et al. found that, for unchanged flow, a lower temperature seemed to be associated with better comfort [45]. Table 1 shows the practical indications of HFNT use in the perioperative setting.

**Table 1:** Suggestions for high flow nasal therapy (HFNT) parameters (flow, FiO_2_, temperature) for preintubation oxygenation and postoperative setting

HFNT	Preintubation	Postoperative setting
Flow	50–60 l min − 1	30–40 l min-1 and increase to match patient’s demand
FiO_2_	1.0	Increase the FiO_2_ until satisfactory SpO_2_ is achieved
Temperature	37°	Titrated to best patient comfort

### Rationale for using HFNT in perioperative medicine

Oxygen is usually provided by a face mask or nasal prongs up to 15 L/min. However, using standard oxygenation methods, as mentioned above, FiO_2_ pharyngeal values may be unstable [23]. Different from conventional oxygen treatments, HFNT may limit air entrainment, enhancing pharyngeal FiO_2_ values [23, 46]**.** Several studies reported HFNT as an effective first-line support for mild to moderate acute hypoxemic respiratory failure [4, 6, 8, 47, 48]. Moreover, HFNT decreased breathing frequency and improved oxygenation [46, 49].

Schwabbauer et al. [50] compared HFNT to NIV and conventional oxygen treatment in functional and subjective respiratory parameters in patients with ARF of hypoxemic origin. They found that HFNT offered an effective compromise between oxygenation and patient comfort and seemed better tolerated than other treatments.

Mauri et al. [21] used a prospective randomized crossover study to evaluate HFNT set at 40 L/min vs. standard therapy (same FiO_2_) for 20-min trials in 15 hypoxemic ARF patients (PaO_2_/FiO_2_ 130 ± 35 mmHg). They assessed the effects of HFNT on arterial blood gases, minute volume (Ve), EELV, patient’s inspiratory effort, ventilation homogeneity and dynamic compliance of the respiratory system. The authors found that HFNT produced several physiological effects in this patient population, including a reduction in inspiratory effort, improved lung volume and dynamic compliance.

The same authors [44] performed a prospective randomized crossover study that compared a standard facial mask with HFNT delivered at different flow rates (30, 45 and 60 L/min) in 17 hypoxemic non-intubated patients with PaO_2_/FiO_2_ ≤ 300 mmHg. They found that increasing the HFNT flow rate to 60 L/min progressively improved lung aeration, dynamic compliance and oxygenation and decreasing inspiratory effort. Interestingly, most of the physiological effects, such as CO_2_ clearance, were already found at the lowest flow rate.

The effects of CPAP (5 cmH_2_O) and HFNT (60 L/min) compared to conventional oxygen therapy were evaluated in 12 ICU patients with hypoxemic ARF [38]. Data were collected during randomly assigned periods of HFNT and CPAP. Each trial lasted approximately 20 min. The authors found no differences in inspiratory effort and in the respiratory rate between HFNT and CPAP. PaO_2_/FiO_2_increased significantly with HFNT compared to conventional oxygen therapy (167.2 [157.2–183.8) vs. 155.8 [109.6–171.3], *p* < 0.01). However, the PaO_2_/FiO_2_ ratio was significantly greater during CPAP than during HFNT. Dyspnea improved with HFNT and CPAP but not significantly. Interestingly, when the authors evaluated patient comfort, they did not find any difference in the trials.

### HFNT use for endotracheal intubation

Endotracheal intubation (ETI) is a potentially life-threatening procedure in the operating room, especially in critically ill patients who require surgery. ETI may expose these patients to the risk of severe desaturation, eventually resulting in cardiac arrest and death [51–53].

Pre-oxygenation prior ETI is a mandatory and crucial step that extends apnea time and delays the eventual desaturation. Oxygenation through a facial mask is generally recommended in patients with healthy lungs prior to general anesthesia [46]. However, hypoxemic patients are prone to severe oxygen desaturation due to their underlying diseases or clinical conditions, which reduce oxygen stores and increase oxygen consumption [52] (e.g., patients with severe obesity, COPD, idiopathic pulmonary fibrosis, neuromuscular diseases, pregnancy). Alone or in combination with HFNT, NIV has been shown to improve oxygenation and to prolong apnea time without desaturation before intubation [15, 54]. The effect of oxygen administration before ETI is to increase body oxygen stores by replacing nitrogen in the FRC. Oxygen administration during apnea after the induction of general anesthesia may theoretically prolong apnea without desaturation. The mass flow of oxygen delivered to the upper airway is driven to alveoli by the pressure gradient generated by continuous oxygen uptake [55]. However, some patients undergoing ETI for general anesthesia may benefit from respiratory support to reduce intubation-related oxyhemoglobin desaturation or in the attempt to delay it [56, 57].

**Fig. 1:**
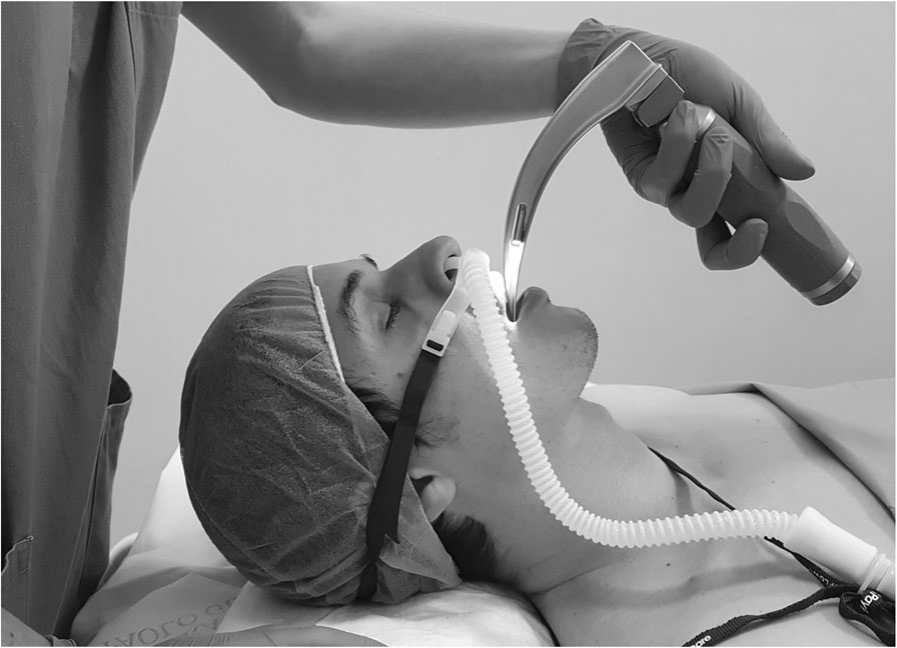
Example of HNFT use for peri-intubation apneic oxygenation. Authors’ own figure

Simple supplemental oxygen administration prior to and/or during ETI cannot be considered a form of ventilator support. NIV reduces alveolar collapse and the likelihood of atelectasis formation, which are responsible for hypoventilation, increased perfusion-ventilation mismatch and eventual hypoxemia [58]. NIV may be used for pre-oxygenation [15], but it must be discontinued during laryngoscopy. Therefore, NIV cannot completely prevent desaturation during tracheal intubation. Owing to the use of dedicated nasal cannulae, HFNT does not interfere with laryngoscopy (Fig. 1). Therefore, HFNT can be used to supply oxygen during peri-intubation apnea [53].

HFNT was also evaluated in 50 patients who were at risk of difficult intubation during awake fiber-optic intubation. HFNT use was safe, well tolerated, and improved oxygenation while avoiding hypercapnia [59]. Heinrich et al. found that HFNT was an effective and safe pre-oxygenation method in a randomized controlled study in 33 obese patients undergoing bariatric surgery [60]. Three pre-oxygenation techniques were tested before rapid sequence intubation (RSI): 1) HFNT set at 50 L/min at a FiO_2_ of 1; 2) CPAP set at 7 cmH_2_O and FiO_2_ of 1; and 3) traditional facial mask at 12 L/min with an FiO_2_ of 1. The primary outcome was PaO_2_ values at defined time points during pre-oxygenation (1, 3, 5, 7, and 8 min). HFNT significantly improved oxygenation during the pre-oxygenation at 5 and 7 min prior to RSI compared to traditional oxygen therapy, but no significant difference in oxygenation compared to CPAP was observed.

Although CPAP should increase EELV by recruiting collapsed alveoli, its beneficial effect might not be associated with an increase in oxygenation [57]. Thus, alveolar recruitment may not be associated with functional recruitment, as defined by improved gas exchange [61].

Patel et al. [62] showed that a transnasal humidified oxygen supply system [transnasal humidified rapid-insufflation ventilatory exchange (THRIVE) technique], which is an analogue of HFNT, can prolong apnea time in patients with anticipated difficult airways who were scheduled for general anesthesia. Patients with obstructive sleep apnea (OSA) undergoing surgery for laryngotracheal stenosis or vocal cord pathology were treated with THRIVE at 70 L/min for 10 min before anesthesia induction and laryngoscopy. THRIVE at 70 L/min was maintained for the entire maneuver until a safe airway was eventually achieved regardless of the number of intubation attempts or the presence of expected or unexpected difficult intubation. The authors found that in patients at risk of difficult airway management, THRIVE increased the apnea time to an average time of 17 min without SpO_2_ levels below 90%. No patients presented adverse events related to CO_2_ toxicity, such as episodes of cardiac arrhythmias.

Raineri et al. assessed the efficacy and safety of HFNT in 45 patients receiving RSI for urgent abdominal surgery. HFNT at 60 L/min and FiO_2_ of 1 was used for 4 min prior to induction and maintained until ETI [53]. Heart rate, SpO_2_, and mean arterial pressure were assessed at baseline (T0), after 4 min on HFNT (T1), during laryngoscopy (T2) and at the time of ETI (T3). Episodes of SpO_2_ values < 3% from baseline were recorded. SpO_2_ increased significantly at T1, T2 and T3 compared to T0. Minimal SpO_2_ was 96%. No episodes of SpO_2_ values < 3% from baseline were found in any patient. Maximum apnea time was 12 min. Mean end-tidal CO_2_ (ETCO_2_) at the time of ETI was 36 mmHg.

Indeed, in critically ill patients there are discordant results on the efficacy of HFNT before intubation [63–66]. The reasons for these results may be the different indications for ETI and the patient’s baseline oxygen value [63].

A recent randomized controlled trial (OPTINIV) evaluated the combination of HFNT at 60 L/min and FiO_2_ 1 coupled with NIV compared to NIV alone as a pre-oxygenation method to reduce desaturation prior to ETI in ICU patients with severe hypoxemic ARF [54]. The primary outcome was the lowest SpO_2_ during ETI. The lowest SpO_2_ values during ETI were significantly higher [100 (95–100)%] in the HFNT + NIV group compared to the NIV alone group [96 (92–99)] (*p* = 0.029).

However, all these data were collected from ICU patients. Extrapolation to patients in the operating room is not certain.

### HFNT in the postoperative setting

General anesthesia, surgery duration and postoperative pain may determine important modifications in the respiratory system. General anesthesia and neuromuscular paralysis induce muscle relaxation with a cranial shift of the diaphragm. This shift reduces pulmonary volumes and induces atelectasis [67, 68]. Modification of lung volume is mainly caused by the reduction of the FRC. Its reduction increases as the surgical site approaches the diaphragm (chest and upper abdomen) [69]. Typically, atelectasis occurs in the sloping portions of the lung next to the diaphragm and may involve nearly 10% of the lung and persist up to 24–48 h after major surgery [67, 70]. After formation, atelectasis may persist up to 24–48 h with use of neuromuscular blocking agents, even in patients undergoing general anesthesia with normal lungs.

Oxygen administration is by far the most common therapy after extubation to correct hypoxemia. During the postoperative period, NIV use may be difficult due to the lack of a proper setting or resources (e.g., in general surgical wards) or because the patient cannot tolerate the interface [71]. Because of these multiple effects, HFNT would seem to be an interesting method to contrast hypoxemia and possibly prevent post-extubation atelectasis, though this remains to be confirmed [72–74]. HFNT may offer a less traumatic interface and potentially offers fewer burdens for the patient and caregiver than NIV. However, the primary limitation of HFNT use is the correction of hypoxemia using high oxygen flow but without correcting substrates, such as atelectasis, due to low pressure levels. HFNT use should find its rationale as a “stand alone” or intermediate level of respiratory support that is in between NIV and standard oxygen therapy or in patients who show intolerance to NIV treatment.

A multicenter randomized trial was performed in 527 mechanically ventilated ICU patients with a low risk of reintubation (251 post-elective or urgent surgery) [75]. The authors randomized patients to receive HFNT (treatment group) or conventional oxygen therapy (control group) after extubation to evaluate the reintubation rate. The study found that HFNT was associated with a reduced risk of reintubation compared to standard oxygen reduced (4.9% versus 12.2%) [75]. However, only half of the included patients were surgical, and it is difficult to reach a conclusive clinical message on this topic.

A further multicenter randomized non-inferiority study compared HFNT and NIV to evaluate the incidence of post-extubation ARF and reintubation in 604 high-risk patients [76]. A total of 232 of the included patients were evaluated after scheduled or urgent surgery. Patients were randomized to receive HFNT or NIV for 24 h after extubation. Sixty-six patients treated with HFNT did not require reintubation (22.8%) vs. 60 (19.1%) treated with NIV. Very interestingly, 42.9% patients in the NIV group vs. none in HFNT group presented adverse effects that required device removal.

Stephan et al. performed a multicenter, randomized, non-inferiority trial (BiPOP Study) to determine whether HFNT therapy was inferior to NIV (administered as two levels of pressure - BiPAP) for the prevention or treatment of ARF after cardiothoracic surgery [77]. Patients were randomized (*n* = 416) to NIV delivered as pressure support ventilation set at 8 cmH_2_O plus 4 cmH_2_O of PEEP, FiO_2_ 50% administered via face mask for at least 4 h/day or HFNT (*n* = 414) 50 L/min with an FiO_2_ of 50%. HFNT was not inferior to NIV since 21% of patients in the HFNT group failed the treatment vs. 21.9% in the NIV group. ICU mortality was not significantly different between the 2 groups. Interestingly, after 24 h, nasal skin lesions were significantly more present in the NIV group.

Futier et al. performed a multicenter randomized trial in 220 patients scheduled for major abdominal surgery (OPERA STUDY) with a moderate to high risk for developing postoperative pulmonary complications (PPCs) and compared post-extubation HFNT versus standard oxygen [73]. The primary outcome was the risk of hypoxemia. Secondary end points were the presence of PPCs within 7 days after surgery, hospital length of stay, and in-hospital mortality. Sixty-two percent of patients from both groups received recruitment maneuvers during lung-protective mechanical ventilation. At 1 h after extubation, the proportion of patients who presented hypoxemia was similar (21% in the HFNT group vs. 27% in the control group). Moreover, the incidence of PPCs between groups over a 7-day postoperative period was not different. The authors found that early application of HFNT vs. standard oxygen after extubation did not reduce the risk of developing pulmonary complications [73]. Interestingly, HFNT was significantly beneficial in the subgroup of patients with complete protective ventilation (receiving high PEEP, low tidal volume and recruitment maneuvers).

## Conclusions

Although most of the evidence on HFNT was collected from ICU studies, HFNT seems to be an interesting noninvasive support in perioperative medicine. HFNT presents some advantages over NIV. HFNT does not require patient cooperation, but it is generally better tolerated and easier to use and requires less equipment and lower nursing workload. On the other hand, since the patient does not need cooperation, surveillance by physicians must be highly reinforced to avoid delays intubation [19]. In addition, HFNT therapy may not the best option for all patients with or who are at risk of postoperative ARF [63]. To date, NIV should be considered the first-line ventilatory support for several perioperative clinical situations because it generates a real assistance on respiratory muscles, as opposed to HFNT therapy. Therefore, NIV should be more indicated in more severe patients, but its role is now discussed in hypoxemic patients [12, 47, 48, 78].

The role of HFNT in postoperative ARF remains an open question with some important issues to be solved, such as which patient will benefit, the correct timing of treatment application and escalation. Further studies are needed to define these important issues in perioperative medicine.

## Abbreviations

ARDS: Acute respiratory distress syndrome
ARF: Acute respiratory failure
ARF: Acute respiratory failure
CO_2_: Carbon dioxide
COPD: Chronic obstructive pulmonary disease
CPAP: Continuous positive airway pressure
EELV: End-expiratory lung volume
EPAP: Expiratory positive airway pressure
ETCO_2_: End-tidal carbon dioxide
ETI: Endotracheal intubation
FiO2: Fraction of inspired oxygen
FRC: Functional residual capacity
HFNT: High flow nasal therapy
ICU: Intensive care unit
NIV: Noninvasive ventilation
OSA: Obstructive sleep apnea
PEEP: Positive end-expiratory pressure
PLB: Pursued lips breathing
PPC: Postoperative pulmonary complication
RH: Relative humidity
RSI: Rapid sequence intubation
SpO_2_: Peripheral oxygen saturation
THRIVE: Transnasal humidified rapid-insufflation ventilatory exchange
Ve: Minute volume
